# Process evaluation of a randomised controlled trial - prevention of sickness absence through early identification and rehabilitation of at-risk patients with musculoskeletal disorders (PREVSAM)

**DOI:** 10.1186/s12913-024-11758-7

**Published:** 2024-10-26

**Authors:** Annika Ekhammar, Maria EH Larsson, Karin Samsson, Susanne Bernhardsson

**Affiliations:** 1 Unit of Physiotherapy, Department of Health and Rehabilitation, Institute of Neuroscience and Physiology, Sahlgrenska Academy, University of Gothenburg, Gothenburg, Sweden; 2 Primary Care Rehabilitation, Region Västra Götaland, Närhälsan Eriksberg, Gothenburg, Sweden; 3 Education, Development and Innovation, Region Västra Götaland, Primary Health Care, Research, Gothenburg, Sweden; 4 Capio Ortho Center Gothenburg, Gothenburg, Sweden

**Keywords:** Rehabilitation, Implementation, Process evaluation, Primary care, Musculoskeletal pain

## Abstract

**Background:**

Musculoskeletal disorders are commonly treated in primary healthcare and may, if not treated adequately, entail a risk for long-term disability and sickness absence. A team-based rehabilitation intervention (PREVention of Sickness Absence for Musculoskeletal disorders, PREVSAM) was evaluated in a randomised controlled trial. The purpose of this study was to evaluate the process of implementing the PREVSAM model in primary care rehabilitation.

**Methods:**

This process evaluation was conducted alongside the trial, collecting quantitative and qualitative data to evaluate how the PREVSAM model was implemented, mechanisms of impact, and contextual factors. Acceptability, feasibility, appropriateness, adaptations, training and support, resources, recruitment, reach, retention, dose, fidelity, and readiness for change were investigated. Qualitative data were collected from healthcare professionals and patients.

**Results:**

Eight of 22 invited rehabilitation clinics (36%) and 28 of 54 healthcare professionals (52%) were included in the PREVSAM trial and this process evaluation. Of 507 eligible patients, 261 (51%) were included. Of those, 134 were randomised to the intervention and 129 (96%) were retained. Twelve healthcare professionals and 15 patients participated in the qualitative evaluations. The model’s essential components; individual assessments and structured, team-based rehabilitation with clear division of responsibilities agreed in a joint health plan; were generally delivered according to protocol. The optional components early access to psychological treatment and workplace contact were delivered to a lesser extent. Perceived acceptability, feasibility, and appropriateness of the PREVSAM model were moderate to high. Several contextual barriers, in the form of missing prerequisites, affected the implementation. Qualitative data showed that the model, with its holistic view, was appreciated by both healthcare professionals and patients.

**Conclusions:**

This process evaluation suggests that PREVSAM is acceptable, feasible and appropriate for patients with MSDs reporting psychological risk factors associated with increased risk for sickness absence. While essential components were implemented with fidelity for most patients, optional components were not. This variability reflects the complexity of the model, its mandatory and optional components, contextual barriers, and the person-centred approach meeting individual patient needs. As all model components were not delivered to all patients, the intervention may have been too similar to treatment as usual to detect differences on a group level. A limitation of the study is that half of the participating rehabilitation clinics withdrew prematurely.

**Supplementary Information:**

The online version contains supplementary material available at 10.1186/s12913-024-11758-7.

## Background

Musculoskeletal disorders (MSDs) are a leading cause of physical disability and productivity loss [1]. About 10-20% of patients with MSDs may develop persistent problems and MSDs are a common reason for sickness absence [2–4]. In Sweden, patients with MSDs are mostly managed in primary care, and make up approximately 60% of patients seeking primary care [5]. Direct access to physiotherapist, i.e., without referral, has been implemented in primary care with positive health effects [6–9]. Early access to physiotherapy treatment has been shown to be generally beneficial, and screening for psychological risk factors is recommended [9–11]. Since the primary care reform in 2018, primary care in Sweden is tasked by the government to not only provide treatment but also to prevent illness [12]. The purpose of the Swedish reimbursement model, “Care choice rehabilitation” [13], is to strengthen the individual’s influence on their own care by offering a free choice of rehabilitation clinics within primary care rehabilitation in Region Västra Götaland. According to the model, care provided should be based on the patients’ individual needs and preferences, be easily accessible, welcoming, offer participation, and have a holistic view. However, there is still a knowledge gap concerning which strategies for MSDs within primary care rehabilitation are the most effective in preventing long-term problems and sickness absence. With the aim to early identify at-risk patients by screening for psychological risk factors and prevent the development of persistent MSD and sickness absence, a team-based rehabilitation model, *Prevention of sickness absence through early identification and rehabilitation of at-risk patients with musculoskeletal disorders*, PREVSAM, was designed [14]. The model was evaluated in a randomised controlled trial (RCT) during 2019–2022 [15]. This process evaluation reports the implementation process and is tightly integrated with the RCT in which effects of the model on patients’ sickness absence and on patient-reported outcomes were measured.

The value of a process evaluation alongside trials is recognised, with the goal to provide insight into how and why the intervention is effective or not, and to help distinguish whether it is the intervention itself or the way it was implemented that has been successful or unsuccessful [16, 17]. Medical Research Council (MRC) guidance recommends that a process evaluation is based on three themes: implementation, mechanisms of impact, and the context [16]. Complex interventions usually need to be adapted when implemented in different contexts [16], and exploring what was delivered in practice may enable the capture of implementation fidelity [18]. This balance between fidelity and adaptation may be handled in different ways by healthcare professionals, and it is important to investigate whether the model components assumed to make the intervention effective are preserved [19]. Other important implementation outcomes are how the intervention was perceived by those delivering it and those receiving it. Capturing their perceptions of adoption, appropriateness, feasibility, and acceptability of the intervention is recommended [20]. Organisational readiness for change refers to how willing and able the professionals are to implement organisational change. It is a complex matter as there are different individual reasons for why a change is valued, but most important to successfully implement a change is that the professionals collectively value the change enough to be motivated to act [21].

## Methods

### Aim

The purpose of this study was to evaluate the process of implementing the PREVSAM model in primary care rehabilitation.

The main research questions were:

- Which components of the PREVSAM model were implemented, and how?
- Which mechanisms of impact were identified?
- Which contextual factors affected the implementation?

### Design

This process evaluation of the RCT in which the PREVSAM model was evaluated, was conducted in accordance with the MRC guidance [16]. Implementation, mechanisms of impact, and contextual factors were investigated, employing quantitative and qualitative methods.

The PREVSAM trial has been described in detail in the study protocol [11] and at ClinicalTrials.gov; Protocol ID: NCT03913325, initial release 12/04/2019. Results of short-term effects of the trial on sickness absence and clinical outcomes have been reported separately [15]. Data on long-term effects of the trial on sickness absence and clinical outcomes and on patients’ experiences have been collected and will be reported in future publications.

### Setting

This study was conducted in two Swedish regions. Region Västra Götaland, with about 1.7 million inhabitants, is Sweden’s second largest region in which Sweden’s second largest city is located. Both public and private rehabilitation clinics within the “Care choice rehabilitation” model are obliged to provide both physiotherapy and occupational therapy. In this model, financial compensation is provided from the regional healthcare authority based on type and number of healthcare provider contacts. Region Värmland is Sweden’s eighth largest region by surface area with about 283 000 inhabitants and has a compensation system based on fixed remuneration with performance requirements.

### Intervention: the PREVSAM model

**Fig. 1:**
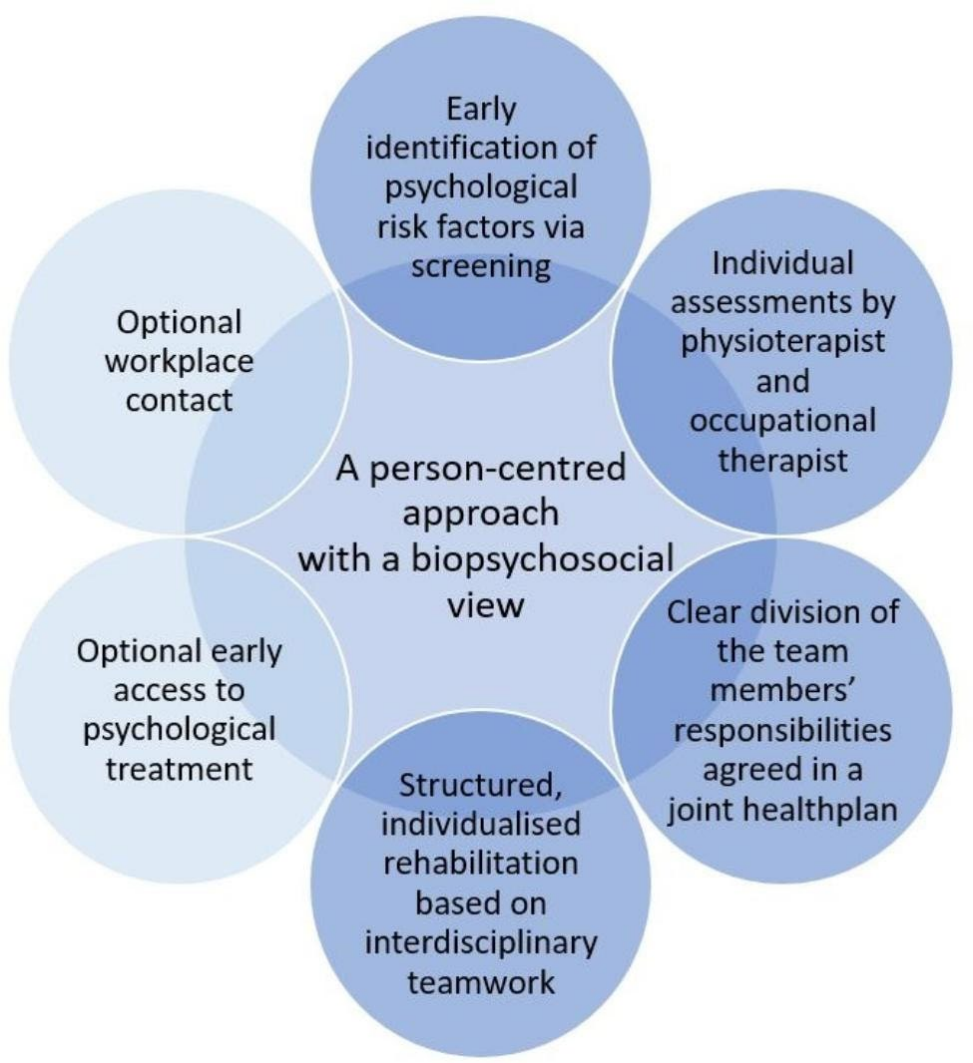
The PREVSAM model’s components

The PREVSAM model was designed to early identify psychological risk factors and provide team-based structured rehabilitation for patients with MSDs, based on a biopsychosocial understanding of pain and using a person-centred approach with clear division of responsibilities (Fig. 1).

To identify patients at risk for developing long-term pain and sickness absence, scoring 40 points or more on the ÖMPSQ-SF was used as a cutoff. Inclusion and exclusion criteria are described in detail in the study protocol [14]. All patients randomised to the PREVSAM model were to be assessed individually by both a physiotherapist and an occupational therapist, followed by a team meeting with the two healthcare professionals and the patient. The team creates a partnership where all three individuals are considered team members. In the team meeting, a joint health plan is established based on the patient’s individual needs and preferences and the PREVSAM team healthcare professionals’ knowledge. In the health plan, the patient’s goals and milestones inspired by the “SMART” approach [22], i.e., be specific, measurable/meaningful, achievable/activity-based, realistic/relevant, and timed, are documented, together with a clarification of responsibilities and which optional treatments available in the PREVSAM model are planned. Early access to psychological treatment can be provided and if so, the psychotherapist is included in the team. Furthermore, work-related factors are mapped and a team dialogue with the patient’s workplace can be initiated. The team members should evaluate the health plan regularly. Rehabilitation should comprise commonly used evidence-based treatments and be structured and coordinated by the PREVSAM team healthcare professionals, but was not specified further. Treatments provided do not need to differ from treatment as usual, except for a structured team-based delivery and the possibility of additional treatments.

### Control: treatment as usual

Treatment as usual for MSDs in primary care rehabilitation is mainly unimodal and comprises physiotherapy treatment, and, to a lesser extent, occupational therapy. Based on their clinical assessment and available resources, the physiotherapists and/or occupational therapists provided rehabilitation comprising commonly used evidence-based treatments. Early identification of risk factors, teamwork, collaboration with psychotherapist/psychologist and the workplace are not standard approaches.

### Implementation of the PREVSAM model

#### Training and support

The first author (AE) was in her role as one of two project coordinators in the PREVSAM trial responsible for training the participating PREVSAM team healthcare professionals in the components of the PREVSAM model. An introduction was scheduled for one and a half hour and was based on “the PREVSAM trial binder”, containing routines for the whole workplace regarding screening, inclusion and exclusion criteria, and patient information. Forms to document visits, including diagnosis and procedure codes, guidance for a person-centred approach and for establishing a joint health plan were also discussed and provided in the PREVSAM trial binder. Due to parental leave and retirement, there were staff changes in some teams, whereas other teams maintained the same PREVSAM team healthcare professionals throughout the trial. New PREVSAM team healthcare professionals received individual training by the project coordinator before replacing their colleagues.

Training was provided in four workshops; the first shortly after the trial started up in 2019, and three more during 2019 and 2020. The aim was to preserve the PREVSAM model’s components, address difficulties, and discuss solutions with the principal investigator, the project coordinators, and with PREVSAM team healthcare professionals from other rehabilitation clinics including the psychotherapist. Each workshop lasted one day. The workshops included lectures on person-centredness, work-related laws and regulations, pain research, patient cases, practical exercises, and time for questions and discussions about the PREVSAM model.

Continuous support to the participating PREVSAM team healthcare professionals was provided by the first author in her role as project coordinator. She was available by e-mail or telephone to answer questions or discuss matters regarding the trial. She also provided information in a monthly e-mail newsletter, which included news about the trial and ongoing recruitment and relevant articles on MSD rehabilitation.

### Outcomes and data collection

Key components of the process evaluation are described in Table 1. Data were collected continuously as the clinics were recruited and the intervention was launched. Outcomes investigated were recruitment, reach, retention, dose, fidelity, training and support, contextual factors, adaptations, and resources. Data on recruitment, reach, and retention were collected, as appropriate, on rehabilitation clinic level, healthcare professional level, and patient level (participants randomised to the intervention in the PREVSAM trial). Dose delivered or received were collected on healthcare professional level and patient level, respectively, and fidelity on healthcare professional level. In view of a possible wider implementation of the PREVSAM model, healthcare professionals’ readiness for change was assessed, in which also acceptability, adoption, appropriateness, feasibility and cost-effectiveness were captured. The data were collected from field notes, study records, questionnaires, attendance lists, and in focus groups and interviews.

**Table 1:** Layout of the process evaluation

Medical Research Council guidance	Key components	Data collection
**Implementation**		
* Which components of the PREVSAM model was implemented and how*, *which training*, *and support were provided?*	1. Recruitment (procedures to approach and attract rehabilitation clinics, healthcare professionals and participants) 2. Reach (number and proportion of rehabilitation clinics, healthcare professionals and participants that participated) 3. Retention (number and proportion of rehabilitation clinics, healthcare professionals and participants that remained) 4. Dose (delivered or received) 5. Readiness for change 6. Fidelity (the extent to which the intervention was delivered as planned) 7. Training and support	Field notes, study records including procedure codes, questionnaires, and attendance lists.
**Mechanisms of impact**		
* How is the PREVSAM model intervention perceived to produce change?*	Healthcare professionals’ experiences and perceptions	Focus group discussions
	Patients’ experiences	Interview study
**Context**		
* How do contextual factors affect the implementation and outcomes?*	1. Contextual barriers and facilitators 2. Adaptations to fit the context 3. Resources	Qualitative and quantitative data. Collaboration managers, healthcare professionals and research group. Reimbursement to participating clinics.

#### Recruitment, reach and retention

The research team collected data on rehabilitation clinic level and on healthcare professional level and documented contacts made in the recruitment process, participation, and training in the intervention. The PREVSAM team healthcare professionals documented and collected data on patient level.

##### Rehabilitation clinics

the number of rehabilitation clinics who agreed to participate. Number of rehabilitation clinics who completed the intervention training, number of those who participated in the intervention, and number of those who fulfilled or left the trial prematurely.

##### Healthcare professionals

the number of physiotherapists, occupational therapists and psychotherapists/psychologists who agreed to participate in the intervention and were trained in the PREVSAM model, and number who participated as PREVSAM team healthcare professionals in the intervention. Recruitment of PREVSAM team healthcare professionals, i.e., physiotherapists and occupational therapists, was done by managers at each participating clinic. In the PREVSAM trial, the psychotherapist was employed by the PREVSAM project and served all participating clinics.

##### Patients

the number of patients who were identified as eligible, number of eligible patients who consented to participate in the study, and number of those who participated in the intervention. Recruitment and retention rate (proportion of eligible patients recruited and retained).

#### Dose

Dose was operationalised as the number or proportion of model components delivered by the PREVSAM team healthcare professionals and/or received by the patients. Data were collected from reports from the PREVSAM teams’ physiotherapists and occupational therapists, including number of team meetings and individual consultations, length of rehabilitation period, and contacts made with the participant’s workplace and with the psychotherapist. The reports included procedure codes registered describing patient-oriented actions for each patient at each consultation [23]. Categorisation of procedure codes was done with guidance from earlier research [24]. Data on number of individual psychological consultations patients attended were collected from the psychotherapist.

#### Readiness for change

Readiness for change can be used as a determinant of implementation success and to identify, and later address, specific deficits in readiness [21], and measuring changes in readiness over time may be useful in assessing factors supporting the intervention delivery [25]. The PREVSAM team healthcare professionals’ beliefs about prevention of chronicity and sickness absence due to MSD, about the PREVSAM model, and their readiness for implementing the model were assessed before and after the intervention using a project-specific questionnaire tailored to the intervention (Supplementary file 1). The questionnaire was designed by the research team, inspired by the recommendations by Weiner et al. [21]. In this questionnaire, “change” was considered to be working according to the PREVSAM model. By assessing the same beliefs after the trial, we aimed to capture whether readiness for change differed after having delivered the PREVSAM intervention. Findings will be discussed in relation to acceptability, adoption, appropriateness, and feasibility - outcomes suggested by Proctor et al. [20] to be indicators of successful implementation. The healthcare professionals had undergone introduction training, but not started inclusion of patients, before answering the questionnaire pre-intervention. They then answered the questionnaire again post-intervention. The questionnaire included 13 items that were answered on 5-point Likert-type scales. Ten items had response options from “completely disagree” to “completely agree”. The middle category was labelled “neither agree nor disagree/do not know”. One item on self-efficacy to work according to the PREVSAM model, was rated from “not at all confident” to “very confident”, with the middle category labelled “neither confident nor unconfident”. One item on general readiness for working with the PREVSAM model, was rated from “not at all ready” to “very ready”, with the middle category labelled “neither ready nor not ready”. One item concerned how rehabilitation according to the PREVSAM model would affect the number of patients at the clinic who could be prevented from entering chronicity and long-term sickness absence. Responses to this item was 1, “fewer patients can be prevented from entering chronicity; 2, “the number will not be affected”; 3, “more patients can be prevented from entering chronicity”; and 4, “do not know”. Wording was slightly adjusted in the post-intervention readiness assessment, reflecting that the healthcare professionals at that timepoint had actually worked with the PREVSAM model.

#### Implementation fidelity

To determine to what extent the PREVSAM model’s components were delivered in the intervention as intended, implementation fidelity was assessed [19]. Fidelity to the PREVSAM model was measured using five self-report items, distributed to the healthcare professionals who participated in the intervention group in conjunction with the post-intervention readiness assessment. The items concerned the extent to which the components of the PREVSAM model had been used. They were answered on 5-point Likert-type scales, with response options from “completely disagree” to “completely agree”.

#### Experiences of the model and perceived contextual barriers and facilitators

Data concerning the PREVSAM team healthcare professionals’ experiences of working according to the PREVSAM model and patients’ experiences of rehabilitation according to the PREVSAM model were collected in focus group discussions and individual interviews, respectively, and reported separately. Four focus groups including twelve PREVSAM team healthcare professionals who had worked according to the PREVSAM model with at least five patients were held [26]. Individual interviews with 15 patients who had been randomised to the PREVSAM intervention were conducted (Ekhammar et al., submitted).

#### Adaptations

In the PREVSAM trial, at-risk patients were identified by the screening questionnaire Örebro Musculoskeletal Pain Screening Questionnaire Short Form (ÖMPSQ-SF) [27]. How and when the screening questionnaire ÖMPSQ-SF could be introduced to the patients was adapted based on the rehabilitation clinics’ usual working methods. Some clinics only had drop-in for the first visit while others only had scheduled first visits. At each rehabilitation clinic, the manager, the PREVSAM team healthcare professionals, the principal investigator, and the project coordinators found solutions for how to carry out the screening. Together they developed routines for how to inform eligible patients about the study and how to report those who agreed to participate to the research team.

#### Resources

During the PREVSAM trial, the participating clinics were reimbursed from the study funds for loss of wages and lost income due to loss of production due to training or participation in workshops. Moreover, a fixed amount per included patient was paid to compensate the clinic for time spent on the screening and inclusion process, team meetings, collaboration with the psychotherapist, workplace contact, and additional documentation.

#### Mechanisms of impact

**Fig. 2:**
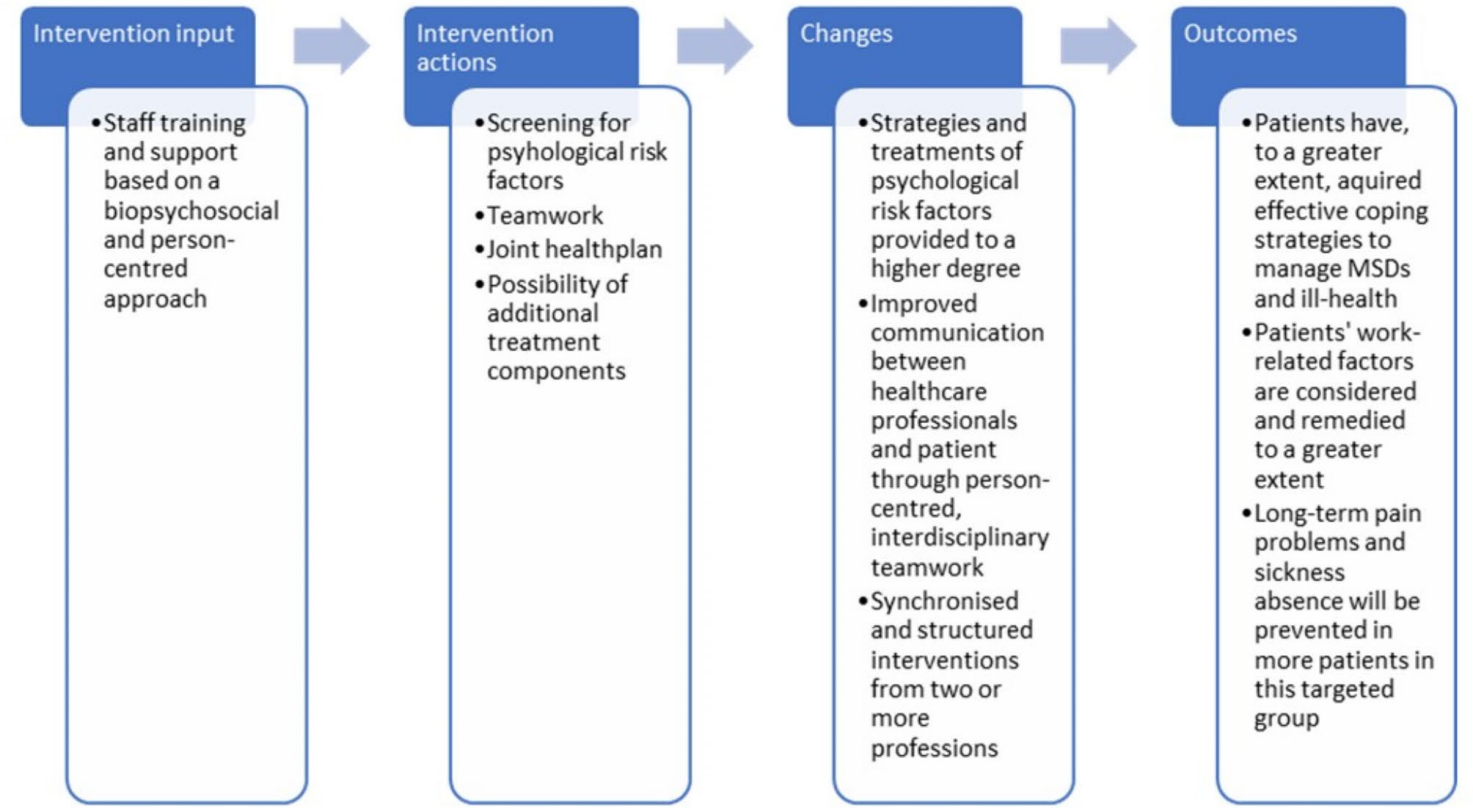
Logic model of the PREVSAM model’s possible mechanisms of impact

Assumptions about how change is to be achieved by rehabilitation according to the PREVSAM model (Fig. 2) are based on our clinical experiences from the field of musculoskeletal rehabilitation and on previous research, as described in our study protocol [14].

##### Data analysis

Quantitative data were analysed with descriptive statistics including frequencies, proportion, number, percentages, means and median values. In the analysis of readiness data, response categories were dichotomised. The response choices “completely agree” and “agree” were dichotomised as positive, and “neither agree nor disagree”, “disagree”, “completely disagree” and “do not know” as negative. The response choices for the items on self-efficacy and readiness to work according to the PREVSAM model, were dichotomised with the response choices “very confident/ready” and “confident/ready” as positive, and “neither confident/ready nor unconfident/not ready” to “not at all confident/ready” as negative. One item concerned how rehabilitation according to the PREVSAM model would affect the number of patients at the clinic who could be prevented from entering chronicity and long-term sickness absence. The response choice “more patients can be prevented from entering chronicity” was interpreted as positive and the choices “the number will not be affected”, “fewer patients can be prevented from entering chronicity; and “do not know” as negative. Fidelity was dichotomised with the response options “completely agree” and “agree” as positive, and the “neither agree nor disagree”, “disagree” and “completely disagree” as negative. Qualitative data were analysed using focus group analysis methodology, and qualitative content analysis [28, 29], respectively.

## Results

### Implementation: what is implemented, and how?

#### Recruitment and attrition

##### Rehabilitation clinics

**Fig. 3:**
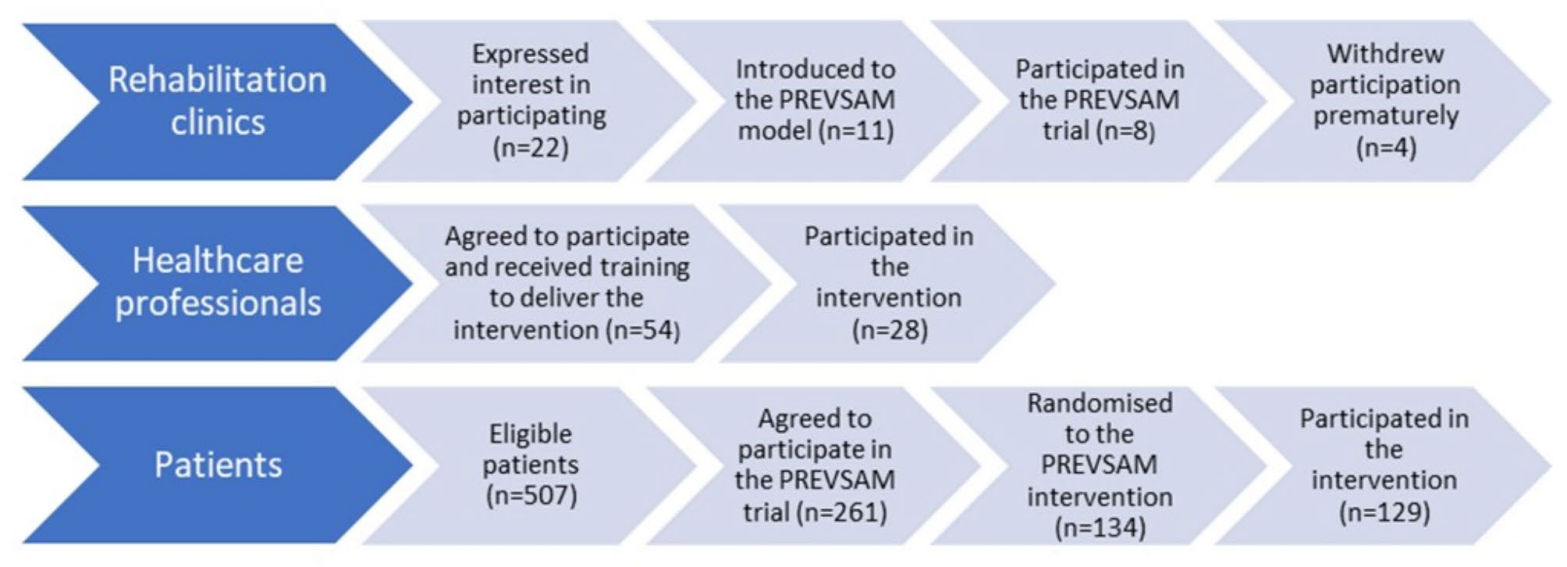
Flow chart of recruitment, reach and retention on rehabilitation clinic level, healthcare professional level, and patient level

The recruitment of participating clinics to the PREVSAM trial started late 2018. Eleven public and 11 private rehabilitation clinics expressed interest in participating. One public and two private clinics declined participation due to the onset of the COVID-19 pandemic, and the principal investigator and the project coordinators visited 19 clinics for further information (Fig. 3). Five public and three private clinics declined to participate due to lack of interest and energy on the part of the staff, even though the managers were positive and encouraged participation. Altogether, staff at five public and six private rehabilitation clinics were trained in delivering the PREVSAM intervention, but the COVID-19 pandemic prevented three of the private clinics to participate due to redundancies amongst staff. In total, eight rehabilitation clinics, seven in Region Västra Götaland and one in Region Värmland, received all training and delivered the PREVSAM intervention. Four clinics were located in rural areas and four in a bigger city, the number of staff varied from about ten to about twenty-five. The first clinic started up in spring 2019 and during the second half of 2019 new clinics started up every month. The last clinic started up during the latter part of 2021.

##### Healthcare professionals

Fifty-four healthcare professionals were trained in the model, of whom 17 physiotherapists, 10 occupational therapists, and one psychotherapist participated in the intervention, i.e., 52%. The main reason for non-participation was working at a rehabilitation clinic that never started up. Four healthcare professionals declined participation for unknown reasons. The number of patients treated according to the PREVSAM model by the physiotherapists and occupational therapists varied between one and 50 per clinic. One of the PREVSAM team healthcare professionals treated 50 patients according to the PREVSAM model, all others treated less than 20, and 14 treated less than five patients each. Twelve of 15 invited PREVSAM team healthcare professionals participated in the qualitative evaluation.

##### Patients

Patients were recruited at their first visit to the included rehabilitation clinics, from May 2019 to June 2022. A total of 507 patients were identified as eligible, and 261 agreed to participate in the study, (recruitment rate 51%). Of those, 134 were randomised to rehabilitation according to the PREVSAM model. Drop-outs were five in total (retention rate 96%). There were large variations between the clinics; from 28 to 78% of identified eligible patients agreed to participate. A higher proportion in the public clinics (60%) agreed to participate than in the private clinics (39%). The recruitment rate was lower than anticipated and varied from 0 to 10 included study patients per week, with a mean of 1.9 during the trial. Fifteen of 21 invited patients participated in the qualitative evaluation.

#### Dose

In the intervention group, 98% of the patients visited a physiotherapist, 81% an occupational therapist, and 86% attended at least one team meeting (Table 2). Treatment length varied from one to 65 weeks, with a mean of 21.9 (SD 14.1) weeks.

**Table 2:** Dose delivered/received of intervention components and length of treatment periods at the rehabilitation clinics (*n* = 129)

	Consultation to physiotherapist	Consultation to occupational therapist	Team meeting	Follow-up by phone	Total number of consultations	Length of treatment period in weeks
Number and proportion of patients who received the intervention component	*n* = 127 98%	*n* = 105 81%	*n* = 111 86%	*n* = 76 59%	*n* = 129	*n* = 125
Mean (SD) (min-max)	7.0 (6.4) (1–54)	1.9 (1.8) (1–14)	1.7 (0.7) (1–4)	2 (1.4) (1–7)	8.4 (7.1) (0–56)	21.9 (1–65)
Median (Q1;Q3)	5 (3;9)	1 (1;2)	2 (1;2)	2 (1;2)	7 (4;11)	19 (13;28.5)

More than 170 different procedure codes were documented. One or more codes were reported at each consultation. Ninety-seven codes were unique, and were grouped into twelve larger categories of interventions and treatment provided (Table 3). The category “Assessment” was the most common intervention, reported for 98% of all patients, followed by “establishing a health plan”, 92%. A joint health plan was distinguished from an individual health plan according to the PREVSAM team healthcare professionals’ reports; joint health plans were reported for 86% of the patients. Procedure codes registered for physical activity, advice and exercise were common, followed by work-related advice and interventions and information and education regarding pain, health, and illness. Behavioural medicine interventions were for example motivational interviewing, stress-management, and different forms of support and training, and physical modalities were treatment of sensory functions and pain, including acupuncture and transcutaneous electric nerve stimulation.

**Table 3:** Assessments and treatments delivered/received, compiled from procedure codes into larger categories (*n* = 129)

Treatment category	Dose delivered/received *n* (%)
Assessment, including screening for psychological risk factors	126 (97.7)
Health plan [of which joint health plan]	118 [111] (91.5,86.0)
Physical activity [advice or exercises]	114 (88.4)
Information and education	81 (62.8)
Work-related advice and interventions	81 (62.8)
Physical modalities	43 (33.3)
Behavioural medicine interventions	37 (28.7)
Body awareness [training and/or consultation]	35 (27.1)
Medical aids [testing, prescribing, training in using and/or follow-up of aids]	24 (18.6)
Patient-related external administration	11 (8.5)
Manual treatment	5 (3.9)

More than half, 54%, of patients requested and were offered psychological treatment, and 40% received the offered treatment (Table 4). A maximum of five sessions with psychological treatment were offered within the PREVSAM intervention, which was what most patients received (mean 4.8, median 5 sessions).

**Table 4:** Dose delivered/received of optional model components (*n* = 129)

Model component	*n* (%)
*Psychological treatment*	
Dose delivered	70 (54.3)
Dose received	
- Attended treatment by PREVSAM psychotherapist	52 (40.3)
- Number of treatment sessions: mean (SD), median (range)	4.8 (0.7), 5 (3)
Delivered but not received:	
The patient cancelled or did not attend scheduled treatment	18 (14)
*Reasons for non-delivery of psychological treatment*	
No need	27 (20.9)
The patient declined	21 (16.3)
The patient already had ongoing psychological treatment	5 (3.9)
Missing data	6 (4.6)
*Work-related interventions*	
Contact with the workplace made by the PREVSAM team healthcare professionals	7 (5.4)
The patients wanted to contact the workplace themselves	13 (10)
Contact already made by the rehabilitation coordinator at the primary care health centre	2 (1.6)
*Reasons for non-delivery of work-related interventions*	
No need	76 (58.9)
*Reasons for non-receipt of work-related interventions*	
The patient did not want to inform the workplace	19 (14.7)
Unspecified other reason	1 (0.8)
Missing data	11 (8.5)

Contact with the workplace after mapping work-related factors was suggested to approximately 35% of patients and contact with the employer was made in 6% of the cases (Table 4).

#### Readiness for change

The healthcare professionals’ self-reported readiness for working according to the PREVSAM model before and after the intervention is presented in Table 5. Pre-intervention, 29 healthcare professionals answered the questionnaire. However, not all of them participated in the intervention. Post-intervention, 18 healthcare professionals answered. All healthcare professionals agreed, both before and after the intervention, that preventing long-term problems and sickness absence is possible and could be cost-effective. Appropriateness and acceptability of the PREVSAM model was high and perceived feasibility was slightly lower; however, the majority felt confident in working according to the PREVSAM model.

**Table 5:** Healthcare professionals’ self-reported readiness for working according to the PREVSAM model pre- and post-intervention

	Proportion who agreed pre- intervention	Proportion who agreed post- intervention	Domain
It is possible to prevent chronicity and long-term sick leave	29/29	18/18	Feasibility
It could be cost effective for the healthcare system to identify persons with MSDs at risk of chronicity and long-term sick leave	29/29	18/18	Cost effectiveness
It could be cost effective for society to identify persons with MSDs at risk of chronicity and long-term sick leave	29/29	18/18	Cost effectiveness
The PREVSAM model could be a possible model for prevention of chronicity and long-term sick leave	29/29	17/18	Appropriateness
Rehabilitation according to the PREVSAM model could prevent more people from chronicity and long-term sick leave.	16/29	17/18	Appropriateness
If the PREVSAM model is effective in preventing chronicity and long-term sick leave I am willing to use it in regular practice	29/29	17/18	Adoption
I feel it is important to work according to the PREVSAM model	27/29	17/18	Acceptability
I feel it is stimulating to work according to the PREVSAM model	28/29	17/18	Acceptability
I feel it is time consuming to work according to the PREVSAM model	27/29	16/18	Acceptability
I feel it is difficult to work according to the PREVSAM model	18/29	10/18	Acceptability
I feel it is about the same as we do today to work according to the PREVSAM model	12/29	11/18	Acceptability
I would like to be able to work according to the PREVSAM model (if prerequisites exists).	not measured	13/18	Acceptability
How confident do you feel in your ability to work according to the PREVSAM model?	21/29	12/18	Feasibility
How ready do you feel to work according to the PREVSAM model?	25/29	14/18	Feasibility

#### Implementation fidelity

The healthcare professionals’ self-reported use of the PREVSAM model components is presented in Table 6. Eighteen healthcare professionals responded. Reasons for non-response were mainly change of workplace, retirement, or parental leave. Most participants reported high fidelity to model components. They reported to a high extent that they used a person-centred approach, established a joint health plan with SMART goals, discussed responsibilities, and that the interventions provided were structured and synchronised. Slightly fewer reported that they revised the health plan if needed. The optional components early access to psychological treatments and workplace contact were delivered to a lesser extent (Table 4).

**Table 6:** PREVSAM team healthcare professionals’ self-reported fidelity to model components (*n* = 18)

Component	Proportion who agreed *n (%)*
I used a person-centred approach, with individualisation of the intervention based on the patients’ resources.	18 (100)
The patient and I formulated SMART-goals/aims together when establishing a joint health plan.	17 (94)
The patient and I discussed their responsibility to follow the joint health plan.	16 (89)
The parts of the intervention and the actions made were structured and synchronised.	16 (89)
If the health plan did not work satisfactory, we revised it together to suit the patients’ needs.	13 (72)

#### Training and support

All PREVSAM team healthcare professionals could not attend all workshops. PREVSAM team healthcare professionals from all rehabilitation clinics participating at the time the workshops were conducted were present at each of the four workshops. Between nine and fourteen attended each workshop. They were encouraged to inform their absent colleagues about the content of the workshops. Nineteen of the PREVSAM team healthcare professionals attended at least one of the four workshops; six attended all four. Some PREVSAM team healthcare professionals were not able to attend any of the workshops for various reasons. All participating PREVSAM team healthcare professionals reported being quite or very satisfied with the workshops at three occasions. On one occasion nine reported being quite or very satisfied and two of eleven reported being neither satisfied nor unsatisfied.

To acknowledge achievements and encourage continued recruitment, the project coordinators and the principal investigator visited the participating rehabilitation clinics on various occasions with cake, lunch, or breakfast, as well as flowers and a new textbook on pain.

#### Mechanisms of impact: how does the delivered intervention produce change?

Qualitative data showed that the PREVSAM model was well received by the PREVSAM team healthcare professionals, as described in the focus groups [26]. They described experiences of a positive impact of working with the model on their work environment and collaboration. They believed that working according to the PREVSAM model was beneficial for patients in need of and motivated for team-based interventions, as well as for their own work situation, with a shared burden and increased joy at work. Working with prevention of sickness absence was seen as leading to benefits for society. The holistic view and the person-centred teamwork were underscored as potential mechanisms of impact, in that it provided a clear framework, widened the range of treatments, and made the patients feel well taken care of. However, the participants presented a less positive attitude towards the screening. Even though early identification of psychological risk factors was perceived as important, the screening was not considered enough to capture patients in need for team-based interventions.

Patients who received rehabilitation according to the PREVSAM model described being grateful for its person-centred, holistic view, and many perceived the opportunity to receive help from several healthcare professions as beneficial for their rehabilitation (Ekhammar et al., submitted). However, there was a large variation in rehabilitation needs amongst the patients, and the model was considered by some to be too comprehensive.

#### Context - how does context affect implementation and outcomes?

Recruitment of the expected number of rehabilitation clinics, especially recruiting clinics from the countryside, was difficult. A high workload and the associated time constraint were perceived as barriers to engage all staff in the screening for eligible patients. The COVID-19 pandemic was another barrier, causing major changes in working conditions with new tasks, a high staff turnover, and patients being discouraged from seeking care that could wait. This might also explain why only about half of those trained in the PREVSAM model participated in the intervention. In addition, some PREVSAM team healthcare professionals went on parental leave; most of them replaced, however replacement was not possible at all clinics.

Adaptations of the PREVSAM model to make it fit the context of primary care rehabilitation were generally not necessary, but some pragmatic adaptations, e.g., how and when to administer the screening questionnaire, were made. Adaptations were also made when it was difficult to provide individual assessments from both PREVSAM team healthcare professionals, or when establishing the joint health plan. These adaptations were primarily due to perceived time pressure and staff sickness absence mainly related to the COVID-19 pandemic, and not because of the model itself. In the focus groups, the PREVSAM team healthcare professionals discussed the need for adaptation if the PREVSAM model were to be implemented. Extended time for the initial patient visit, and for a team meeting with and without the patient was considered necessary, and early access to psychological treatment at the rehabilitation clinic was desirable.

Even though the clinics received financial compensation, the PREVSAM team healthcare professionals reported major challenges to make time for teamwork due to the high workload. The current reimbursement system was experienced as a barrier because team assessments/visits were not fully reimbursed. Other organisational conditions, such as difficulties in prioritising screening and having early access to psychological treatment when needed, were a concern for a potential implementation of the PREVSAM model in ordinary primary care rehabilitation.

## Discussion

The PREVSAM model’s essential components—individual assessments and structured, team-based rehabilitation with clear division of responsibilities agreed in a joint health plan —were delivered to a high degree, suggesting a generally good implementation fidelity. The optional components early access to psychological treatment and workplace contact were delivered to a much lower extent, but this is in line with the model’s person-centred approach. Tailoring the treatments provided to the patient’s needs was advocated in the PREVSAM intervention protocol. Participating healthcare professionals considered the model acceptable, feasible and appropriate for patients with MSDs reporting psychological factors associated with risk for sickness absence, and experienced a positive impact on their work environment and collaboration. Participating patients described the model’s holistic view as facilitating rehabilitation.

Maintaining a holistic perspective in primary care has been suggested as *‘to be informed about a patients’ whole life situation in order to be able to create a sense of security and coherence in the patient’* ([30], p.5). This view is fundamental in the person-centred approach recognising the person behind the patient role [31]. The target population in the PREVSAM trial is not homogenous, underscoring the necessity of a person-centred approach and the tailoring of treatments. Previous research indicates that some patients with MSDs will benefit from unimodal treatment, such as self-management advice, exercise therapy, or psychosocial interventions, while others will only improve with more extensive, multidisciplinary rehabilitation [32, 33]. However, it remains unclear which patients who are most likely to respond to which treatments [32, 33].

Using a screening instrument such as ÖMPSQ-SF to identify the need for more complex treatment, including psychological treatment and communication between patient, employer, and healthcare professionals, is recommended in clinical guidelines [34]. Guidance on how to select patients to screen and the best timing for screening has also been proposed [35]. The patients in the PREVSAM trial reported psychological risk factors that may play a role in the transition to long-term problems and sickness absence, and that need to be addressed in various ways. The PREVSAM team healthcare professionals were positive about screening already at the first visit, but expressed concern that the screening may not fully capture those in need of a more comprehensive rehabilitation. In line with their wish, a more thorough follow-up assessment after screening is suggested to help avoiding both over- and under treatment [35]. Moreover, concerns were raised by the PREVSAM team healthcare professionals that the screening for psychological risk factors may be difficult to implement. The participants believed some colleagues felt overwhelmed if, with an already high workload, they had to address a broader spectrum of illnesses.

Fidelity to the model’s essential components individual assessments and creation of a joint health plan was generally high. However, self-reported fidelity must be interpreted with caution. Goal setting and creating the health plan was done by the PREVSAM team, including the patient, who jointly decided which goals and treatments to include in the health plan to address the identified physical, psychological, and work-related factors. Despite this freedom of choice, we believe that the procedure codes compiled into treatment categories provided pertinent information about implementation fidelity. Again, the results must be interpreted with caution. Treatments may have been given without procedure codes being registered, and codes might have been registered for treatments that were not actually delivered as intended. Fidelity was lower to performing follow-ups and/or revising the health plans. This finding is in line with a review on person-centred goal setting by Kang et al. [36], reporting that many goal-setting intervention components, notably follow-up, often are underimplemented in clinical practice.

Although optional, early access to psychological treatment was an important part of the PREVSAM model. Only slightly more than half of the patients were offered this possibility and only two fifths opted to take advantage of it. This low proportion may be due to psychological risk factors are already addressed in treatments by the PREVSAM team healthcare professionals at the rehabilitation clinics. Physical activity advice or exercises were provided to about 88% of the patients, which not only improves physical function but also addresses mental health. Using physical activity as treatment to reduce depression, anxiety, and stress symptoms is highly recommended [37]. Behavioural medicine interventions were registered for nearly 30% of the patients, indicating that other treatments targeting mental health were used, such as counselling about demands/stress/performance problems and motivational interviewing. However, the low proportion of patients receiving psychological treatment could also be due to it not being perceived as relevant (by the PREVSAM team healthcare professionals and/or the patients). The low implementation fidelity of optional components is an example of the balance that needs to be achieved between fidelity and adaptation, as discussed by Perez et al. [38] in their proposed modified implementation fidelity framework.

The absence of registered procedure codes for mapping work-related factors, in nearly 40% of patients, raises concerns regarding fidelity. The procedure codes do not match the percentage in the study reports, in which they stated that more than 60% of the patients had no need for contact with the workplace, and very few wanted help from them with this contact. Although optional, maybe fewer than we anticipated in this patient group needed help with work-related factors, and therefore those procedure codes were not registered? Low fidelity in regard to workplace contact is consistent with a previous Norwegian study, in which initiating collaboration with employers was reported to be difficult due to uncertainty about what it might entail for both patients and healthcare professionals [39]. A perceived lack of suggestions for concrete work-related interventions for patients with chronic musculoskeletal related pain in the Swedish Rehabilitation Guarantee Guidelines has been reported earlier [40]. Current knowledge supports that multidisciplinary efforts, including healthcare provision, service coordination, and work accommodation components, seem most effective for rehabilitation of patients with MSDs [41, 42]. However, the participants in this study were at risk for but not yet afflicted by chronic pain and sickness absence.

Some possible explanations for why so many participants did not perceive a need for the optional treatments emerged in the qualitative data. Both the PREVSAM healthcare professionals and the patients experienced that the treatments provided at the rehabilitation clinics were mostly sufficient, and for some, it was not the right time to engage in a more comprehensive rehabilitation. Only a few patients reported work-related problems, and, in these cases, they described a preference for making the contact with the workplace themselves or to handle work-related issues without informing their employer. In previous qualitative research, patients have described a wish for advice on self-management and on where to obtain information and support, including for work-related issues [43]. Moreover, they underscored the importance of continuity of care and building a relationship with their healthcare provider, who should know their own professional limitations and when to refer on [43].

Both PREVSAM team healthcare professionals and patients described difficulties with the rehabilitation. Some patients dropped out, did not attend treatment sessions, or did not follow the health plan as agreed. This low treatment adherence could, according to both PREVSAM team healthcare professionals’ and patients’ experiences, be due to mental illness, not a good time in life to make health-related changes, or that the joint health plan was not considered necessary, as well as experienced lack of support from the PREVSAM team healthcare professionals. The implementation of the PREVSAM model may have been complicated by the creation of the joint health plan. In previous research, barriers to person-centred goal setting are suggested to be that not all patients actively want to get involved in setting goals and that staff may find it difficult to work person-centred, due to lack of time and skills [36]. As a result of perceived lack of support and difficulties creating a joint health plan, a few patients may have experienced that, as in the words of Carroll et al. ([44], p.3), ‘an intervention could be delivered but delivered badly’.

Amongst the PREVSAM team healthcare professionals, trust in each other’s competencies was perceived as a facilitator. On the other hand, involving multiple healthcare professions may also, according to some PREVSAM healthcare team members, have unnecessarily complicated the rehabilitation. Previous research did not identify any differences between multidisciplinary treatment and other treatments in reducing sick leave and improving time to return-to work for subacute low back pain [33]. Based on the assumption that a uniprofessional design could be a better choice, the WorkUp study investigated adding early workplace dialogue to physiotherapy treatments, and reported improved work ability in patients with neck or back pain [45]. These findings support the notion that a multidisciplinary approach may be redundant for some patients. However, multidisciplinary treatment improved mental health more effectively compared with usual care [33]. According to both our quantitative and qualitative findings, this patient group may need different pathways of care. The PREVSAM model’s flexibility is helpful but may need to be further developed to capture “the right treatment for the right patient at the right time”.

Although complex in itself, the PREVSAM model’s complexity was further increased by both its level of flexibility in terms of interventions and treatments to be provided, and by the attitudes, expertise and skills required by those delivering and receiving the intervention [18]. Attitudes towards the PREVSAM model were generally positive amongst both PREVSAM team healthcare professionals and patients in the intervention. Acceptability and feasibility were moderate to high and adoption, appropriateness, and perceived cost-effectiveness of the PREVSAM model were high. However, a more ambivalent attitude emerged concerning the willingness to work according to the PREVSAM model in the future. Whether this attitude is related to working according to the model or to working with the target patient group is unclear. To reach the target population, all healthcare professionals at the rehabilitation clinics needed to be involved in the screening and in informing eligible patients about the study. However, the context varied amongst the clinics in terms of recruitment and reach. Some clinics identified a high proportion of eligible patients, of whom 75-80% consented to participate in the trial. When those responsible for delivering the intervention are enthusiastic about it, higher levels of implementation fidelity can be expected [19]. The PREVSAM team healthcare professionals at these clinics worked according to the PREVSAM model with a considerable number of patients and thus gained a lot of experience. Other clinics had more difficulties with identifying eligible patients, and of those only 30-35% consented to participate in the trial.

Despite good methodological quality in conducting clinical trials, description and fidelity of the intervention are often insufficiently reported, and very few studies report on skills acquisition of providers [46, 47]. Non-significant treatment results can potentially be attributed to providers receiving inadequate training in the intervention delivery [47]. Differences in healthcare professionals’ skills and professional experience reflect the clinical reality, but should be considered as they may influence the implementation of a new model. To put it another way; a model can never be better than its practitioners.

Challenges in recruiting healthcare professionals in intervention studies are well known, in that they may be reluctant to participate in a study even when participation is supported by the managers [48]. Our plan was to include ten clinics; each including 40 patients, which we did not achieve. When staff buy-in regarding the decision to participate has not been achieved and the decision is perceived as a top-down instruction, recruiting, and retaining participants are more difficult. Our experience is in concordance with previous research findings that at some clinics the staff with a perceived high workload, contrary to their managers, did not believe that engagement in a trial would be worthwhile or beneficial [21, 48]. Moreover, as suggested by Axén et al. [48], some of the healthcare professionals may not believe that the answer to the research question is vitally important to their organisation, profession, or themselves or that it would make much difference to the way they already practise.

Amongst those who left the trial prematurely, the burden of establishing new routines may have been too demanding, and with a lack of support from colleagues and/or managers they may have lost motivation. High workload and lack of support from managers have been reported in previous research as barriers to implement work-focused rehabilitation [49]. Through our workshops and monthly newsletters, we tried to support the PREVSAM team healthcare professionals and highlight the relevance of conducting the trial. We also rewarded all staff at the participating clinics to encourage their participation, which is also recommended by Axén et al. [48] and was, to our knowledge, appreciated. As the participating rehabilitation clinics received considerable support including financial compensation, the PREVSAM team healthcare professionals believed that implementing the PREVSAM model in routine practice could prove even more challenging without such support. Barriers may then occur at the provider level, from individual staff or team members, and the reluctance may be due to factors at the organisational and policy levels [50].

Just over half of the invited patients accepted to participate in the PREVSAM trial. Most patients included in both the trial and in the interview study were from the same rehabilitation clinic, mainly due to difficulties in recruiting enough clinics and some of the clinics withdrawing prematurely from the trial. The patients may also have thought that the intervention was not interesting, or that participating in additional treatment or answering the questionnaires would be too time-consuming. Patients declining to participate in a study is a common problem in research [48]. During the COVID-19 pandemic, recruitment became even more difficult as patients were advised not to seek care if treatment could wait, and digital appointments were encouraged. Identifying eligible patients for the study was made more difficult during the pandemic due to new directives from health authorities, increased sickness absence, and staff turn-over. Therefore, the recruitment rate was considerably lower than estimated.

### Methodological considerations

Conducting an RCT in a primary care rehabilitation context has many challenges, for instance ensuring that the intervention provided adheres to the protocol when different professions at different locations are to provide the intervention. Toomey et al. [46] suggest that fidelity should be an integral part when conducting trials, especially for pragmatic, complex interventions. Introducing a complex, multi-component intervention based on a new model, such as PREVSAM, is thus challenging. Not only does the model include multiple components, of which some essential and some optional; it also involves multiple professions and multiple types of interventions and treatments. The optional components, as well as the person-centred approach, require by definition certain adaptations and complicate the assessment of implementation fidelity. A certain amount of adaptation to the local context and conditions is likely necessary to achieve high-quality implementation, as is fidelity to essential components. A combination of fidelity and adaptation is particularly relevant when the model/intervention is complex [51], but the optimal balance between fidelity and adaptation remains unclear. The local adaptations made to fit the individual clinics participating in the PREVSAM trial were rather minor and were not likely to influence the study outcome.

This process evaluation reveals a wide range in number of consultations, length of treatment, and registered procedure codes, suggesting that the patients had widely varying needs of support in managing their MSDs. This finding supports previous research stating that qualitative data are necessary to complement quantitative data in a process evaluation [16, 52]. Our qualitative findings generated an understanding of the patients’ perspective of their own healthcare needs and of the PREVSAM team healthcare professionals’ perspective, and provided useful information on how to further develop the PREVSAM model and improve its potential impact on health and sickness absence.

In the PREVSAM project, the process evaluation and outcome evaluation are performed by the same research team. Separating or integrating process evaluation and outcome evaluation and research teams both have advantages and disadvantages [21]. In this study, the second author is the principal investigator of the PREVSAM project, and all authors are involved in different studies within the project. This might raise concerns about researcher bias; whether we as researchers have a professional interest in portraying the intervention positively. On the other hand, it can also be an advantage that we as researchers in all parts of the PREVSAM project are well acquainted with both the practical circumstances, i.e., the role of the context, and the data. Our position is that we have a desire to improve MSD rehabilitation within primary care but do not have any vested interests in whether the PREVSAM model is proven superior or not compared with treatment as usual.

The MRC guidance emphasis that there is no consensus on the definition of process evaluation, and that it may entail many methods [16]. Using an established theoretical framework underpinning causal assumptions on mechanisms of impact may be an advantage. However, we base our assumptions on our experiences from the field of musculoskeletal rehabilitation in clinic and previous research, which, according to MCR guidance, may be a better way than trying to impose an established theoretical framework.

A limitation of this study is related to the readiness assessment pre- and post-intervention. First, we do not know which of those who answered pre-intervention who participated in the intervention and answered post-intervention. Second, the questionnaire was developed for this project, and not subjected to psychometric testing. As the instrument needed to be both context- and intervention-specific, we found no relevant published instrument that we could use. The lack of instruments that has undergone thorough psychometric testing is a known concern [21]. Moreover, insufficient reliability, validity, or practicality of existing measures makes it unclear whether to recommend further use of them and highlights the need for developing high-quality practical measures [53, 54]. Fidelity was also self-reported, and a more objective approach might have increased reliability. Nevertheless, we believe that the findings from the questionnaires used contribute to the process evaluation.

Another limitation, or challenge, was the difficulty to distinguish between dose delivered and dose received, both related to measuring difficulties and uncertainty in what may have caused the difference between the two measures. Did the PREVSAM team healthcare professionals not discuss and offer a model component (did not deliver) or were the patients not interested and declined the offer (did not receive)? It can also be viewed as a limitation that the study on patients’ experiences of treatment according to the PREVSAM model, from which we draw data and conclusions in this process evaluation, has not yet been published.

### Implications for practice and suggestions for further research

The PREVSAM model may be a good option for treating patients with MSDs in whom psychological risk factors are identified and who are in need for and motivated for engaging in a more extensive rehabilitation. However, identified barriers should be addressed before implementing the PREVSAM model in routine practice. The short-term follow-up did not show a statistically significant difference between PREVSAM and treatment as usual at the *p* < 0.05 level [15], but the long-term effects are yet to be evaluated. Further research is warranted on how to early identify those people with MSDs who need more extensive rehabilitation. To investigate how to address identified contextual barriers, qualitative research could be used. For example, exploring managers’ attitudes and suggestions regarding implementation of the PREVSAM model.

## Conclusions

Overall, this process evaluation suggests that the PREVSAM model is an acceptable, feasible and appropriate rehabilitation model for patients with MSDs reporting psychological factors associated with risk for sickness absence. The PREVSAM model’s essential components were generally delivered in accordance with the protocol for most patients, while optional components were delivered to a lesser degree. The model was perceived as beneficial by both healthcare professionals, who experienced a positive impact on their work environment and collaboration, and by patients, who appreciated the model’s holistic view and described feeling seen, taken seriously, and treated respectfully. The essential components of individual assessments and team-based rehabilitation with a joint health plan, were perceived as potential mechanisms for preventing long-term problems and sickness absence. Several contextual factors, including the COVID-19 pandemic, influenced the delivery of the intervention. Notably, half of the participating rehabilitation clinics withdrew prematurely. In addition, not all patients in the target group needed interventions from the whole team. Consequently, the participants not receiving all components in the PREVSAM model may have received similar interventions to those who received treatment as usual. Even though the PREVSAM model’s proposed mechanisms of impact in our logic model were considered important, contextual factors and the broad variation in the individual patient’s need may have led to the PREVSAM intervention being too like treatment as usual in too many patients to detect clinical differences on a group level.

## Supplementary Information

Supplementary Material 1.

## Data Availability

The data are available on reasonable request.

## Abbreviations

MSD: Musculoskeletal disorder
PREVSAM: Prevention of sickness absence through early identification and rehabilitation of at-risk patients with musculoskeletal disorders

## Glossary

PREVSAM team healthcare professionals: Healthcare professionals trained in the PREVSAM model who participated in the PREVSAM trial and worked according to the PREVSAM model
