# Supplementary material for: Process evaluation of a randomised controlled trial - prevention of sickness absence through early identification and rehabilitation of at-risk patients with musculoskeletal disorders (PREVSAM)
Source: BMC Health Serv Res. 2024 Oct 26;24:1286. doi: 10.1186/s12913-024-11758-7 (PMC11515810; doi:10.1186/s12913-024-11758-7)
Supplement: Supplementary file 1 — Supplementary Material 1. [file 12913_2024_11758_MOESM1_ESM.pdf]

Pre-intervention questionnaire

This survey is aimed at you who will participate as an interventional physiotherapist, occupational therapist, and psychotherapist in the PREVSAM trial.

What is your position on the following statements?

1. It is possible to prevent chronicity and long-term sick leave in patients seeking help in primary care for acute and subacute musculoskeletal disorders.
□ completely agree □ agree□ neither agree nor disagree/do not know □ disagree □ completely disagree

2. It could be cost effective for the healthcare system to identify persons with MSDs at risk of chronicity and long-term sick leave
□ completely agree □ agree □ neither agree nor disagree/do not know □ disagree □ completely disagree

3. It could be cost effective for society to identify persons with MSDs at risk of chronicity and long-term sick leave
□ completely agree □ agree □ neither agree nor disagree/do not know □ disagree □ completely disagree

4. The PREVSAM model could be a possible model for prevention of chronicity and long-term sick leave.
□ completely agree □ agree □ neither agree nor disagree/do not know □ disagree □ completely disagree

5. To what extent do you think that rehabilitation according to the PREVSAM model would affect the number of patients at the clinic who could be prevented from entering chronicity and long-term sickness absence
□ more patients can be prevented from entering chronicity □ the number will not be affected □ fewer patients can be prevented from entering chronicity □ do not know

6. If the PREVSAM model is effective in preventing chronicity and long-term sick leave I am willing to use it in regular practice
□ completely agree □ agree □ neither agree nor disagree/do not know □ disagree □ completely disagree

How do you feel about starting to work according to PREVSAM?

7. I feel it is important to work according to the PREVSAM model
□ completely agree □ agree □ neither agree nor disagree/do not know □ disagree □ completely disagree

8. I feel it is stimulating to work according to the PREVSAM model
□ completely agree □ agree □ neither agree nor disagree/do not know □ disagree □ completely disagree

9. I feel it is time consuming to work according to the PREVSAM model
□ completely agree □ agree □ neither agree nor disagree/do not know □ disagree □ completely disagree

10. I feel it is difficult to work according to the PREVSAM model

□ completely agree □ agree □ neither agree nor disagree/do not know □ disagree □ completely disagree
11. I feel it is about the same as we do today to work according to the PREVSAM model
□ completely agree □ agree □ neither agree nor disagree/do not know □ disagree □ completely disagree

12. How confident do you feel in your ability to work according to the PREVSAM model?
□ very confident □ confident □ neither confident nor unconfident □ unconfident □ not at all confident

13. How ready do you feel to work according to the PREVSAM model?
□ very ready □ ready □ neither ready nor not ready □ not ready □ not at all ready

Post-intervention questionnaire

This survey is aimed at you who have worked as a physiotherapist, occupational therapist, and psychotherapist according to the PREVSAM model.

What is your position on the following statements?

1. It is possible to prevent chronicity and long-term sick leave in patients seeking help in primary care for acute and subacute musculoskeletal disorders.
□ completely agree □ agree□ neither agree nor disagree/do not know □ disagree □ completely disagree

2. It could be cost effective for the healthcare system to identify persons with MSDs at risk of chronicity and long-term sick leave
□ completely agree □ agree □ neither agree nor disagree/do not know □ disagree □ completely disagree

3. It could be cost effective for society to identify persons with MSDs at risk of chronicity and long-term sick leave
□ completely agree □ agree □ neither agree nor disagree/do not know □ disagree □ completely disagree

4. The PREVSAM model could be a possible model for prevention of chronicity and long-term sick leave.
□ completely agree □ agree □ neither agree nor disagree/do not know □ disagree □ completely disagree

5. To what extent do you think that rehabilitation according to the PREVSAM model would affect the number of patients at the clinic who could be prevented from entering chronicity and long-term sickness absence
□ more patients can be prevented from entering chronicity □ the number will not be affected □ fewer patients can be prevented from entering chronicity □ do not know

6. If the PREVSAM model is effective in preventing chronicity and long-term sick leave I am willing to use it in regular practice
□ completely agree □ agree □ neither agree nor disagree/do not know □ disagree □ completely disagree

7. I felt it was important to work according to the PREVSAM model

□ completely agree □ agree □ neither agree nor disagree/do not know □ disagree □ completely disagree

8. I felt it was stimulating to work according to the PREVSAM model
□ completely agree □ agree □ neither agree nor disagree/do not know □ disagree □ completely disagree

9. I felt it was time consuming to work according to the PREVSAM model
□ completely agree □ agree □ neither agree nor disagree/do not know □ disagree □ completely disagree

10. I felt it was difficult to work according to the PREVSAM model
□ completely agree □ agree □ neither agree nor disagree/do not know □ disagree □ completely disagree

11. I felt it was about the same as we do today to work according to the PREVSAM model
□ completely agree □ agree □ neither agree nor disagree/do not know □ disagree □ completely disagree

12. How confident do you feel in your ability to work according to the PREVSAM model?
□ very confident □ confident □ neither confident nor unconfident □ unconfident □ not at all confident

13. How ready do you feel to work according to the PREVSAM model?
□ very ready □ ready □ neither ready nor not ready □ not ready □ not at all ready

14. I would like to be able to work according to the PREVSAM model (if prerequisites exists)
□ completely agree □ agree □ neither agree nor disagree/do not know □ disagree □ completely disagree

Further questions regarding working according to the PREVSAM model

I used a person-centred approach, with individualisation of the intervention based on the patients' resources.
□ completely agree □ agree □ neither agree nor disagree/do not know □ disagree □ completely disagree

The patient and I formulated SMART-goals/aims together when establishing a joint health plan.
□ completely agree □ agree □ neither agree nor disagree/do not know □ disagree □ completely disagree

The patient and I discussed their responsibility to follow the joint health plan.
□ completely agree □ agree □ neither agree nor disagree/do not know □ disagree □ completely disagree

The parts of the intervention and the actions made were structured and synchronised.
□ completely agree □ agree □ neither agree nor disagree/do not know □ disagree □ completely disagree

If the health plan did not work satisfactory, we revised it together to suit the patients' needs.
□ completely agree □ agree □ neither agree nor disagree/do not know □ disagree □ completely disagree
